# An open-label, single-arm trial of cryoneurolysis for improvements in pain, activities of daily living and quality of life in patients with symptomatic ankle osteoarthritis

**DOI:** 10.1016/j.ocarto.2022.100272

**Published:** 2022-05-15

**Authors:** Thomas A. Perry, Neil A. Segal

**Affiliations:** a Kennedy Institute of Rheumatology, Centre for OA Pathogenesis Versus Arthritis, University of Oxford, Roosevelt Drive, OX3 7FY, UK; b University of Kansas Medical Center, Kansas City, KS, USA; c The University of Iowa, Iowa City, USA

**Keywords:** Cryoneurolysis, Ankle, Osteoarthritis, Pain, Function

## Abstract

**Objective:**

Cryoneurolysis, cold-induced reversible conduction block of peripheral nerves, is an effective treatment for reducing knee osteoarthritis (OA) symptoms and opioid use following knee arthroplasty. There are however, limited data concerning its use for ankle OA. Our aim was to assess clinically significant long-term symptomatic relief of ankle OA with cryoneurolysis.

**Method:**

This single-center, open-label trial included participants aged >18 years with radiographic tibiotalar OA, unilateral ankle pain ≥5/10 on Numerical Rating Scale (NRS), and with no ankle surgery within 6-months of screening. Following ultrasound-guided cryoneurolysis of nerves in the participant's pain distribution (sural, saphenous, superficial and/or deep fibular nerves), outcomes were assessed at clinic visits (6, 12 and 24-weeks) and by telephone interview (3, 9, 18-weeks). The primary endpoint was change in Foot and Ankle Outcome Score (FAOS) (pain subscale) at 12-weeks. Change in quality of life (FAOS-QoL), activities of daily living (FAOS-ADL), NRS-pain, and physical performance measures were also assessed. Longitudinal mixed models were constructed to evaluate changes from baseline at 6, 12- and 24-weeks post-treatment.

**Results:**

Forty participants enrolled (50% female, mean ​± ​SD age 63.0 ​± ​12.8 years). At 12-weeks post treatment, FAOS-pain (20.8, p ​< ​0.0001), ADL (18.1, p ​= ​0.0003), QoL (19.9, p ​= ​0.0003) and NRS-pain (−2.6, p ​< ​0.0001) were significantly improved from baseline. No difference in 40-m fast-paced walking test was detected at 12-weeks post-treatment (−1.2sec, p ​= ​0.59). For all outcomes, similar findings were observed at 6- and 24-week visits.

**Conclusion:**

Cryoneurolysis resulted in statistically significant improvements in ankle pain, physical function and QoL for up to 24-weeks in participants with unilateral, symptomatic ankle OA.

## 1 Introduction

Osteoarthritis (OA) is a global health concern, a leading contributor to loss of function, disability and painful symptoms [1,2], and affects approximately a third of adults [3]. The prevalence of symptomatic, radiographic ankle OA in community-dwelling adults is estimated at 3.4% [4]. Further, the prevalence of ankle pain has been reported to exceed 7% in general practices among adults in the UK [5]. However, despite a moderate prevalence combined with the disabling nature of ankle OA [6], the development of treatments to manage ankle OA symptoms has been slow. Finding a safe treatment that could alleviate symptoms and improve activities of daily living in people with ankle OA, while avoiding the side effects of pharmacological and surgical therapies, is a key objective.

Osteoarthritis represents the biggest unmet medical need of all musculoskeletal conditions [7] and, despite efforts, there are currently no licensed disease-modifying osteoarthritic drugs (DMOADs) [8]. Subsequently, attention has turned to identifying treatments that best manage symptoms and/or improve function and quality of life. Typically, management of OA involves pharmacologic and non-pharmacologic interventions [9,10].

For ankle OA, rates of pharmacological management have been reported to be much higher than non-pharmacological strategies (e.g. lifestyle advice and allied health referral) [11] with treatment algorithms mirroring procedures for the management of other lower-limb joint sites [12]. There are, however, well recognized adverse side-effects and limitations to both pharmacological and surgical treatments. For instance, whilst NSAIDs have shown moderate effects on OA symptoms [13] with data from a recent meta-analysis of 36 randomised clinical trials reporting an effect size of −0.30 (95% CI -0.40 to −0.20) for pain relief and −0.35 (95% CI -0.45 to −0.24) for functional improvement following 2–12 weeks of treatment [14], NSAIDs have been shown to carry an increased risk of gastrointestinal [15], cardiovascular [16], and renal [17] adverse events. Furthermore, whilst joint replacement has shown to be an effective surgery to manage symptoms, it may not be suitable for all patients [18,19]. Therefore, there is a need to identify therapeutic options that alleviate pain and improve physical function whilst also reducing opioid use and outpatient healthcare costs.

Cryoneurolysis has demonstrated promise as a novel [20], effective therapeutic technique for providing long-term analgesia [21], including for cervicogenic headache [22], neuropathic pain [23,24] and phantom-limb pain [25]. The mechanism of action is well understood and has been described previously [26]. In short, cryoneurolysis creates a reversible conduction block of pain signals by the formation of precise, cold-zone lesions that cause Wallerian degeneration [27] while the nerve bundle remains intact, allowing for complete regeneration and functional recovery [26]. The duration of the pain relief depends on the individual and the distance of the treatment from the terminal axon.

Cryoneurolysis was found to significantly reduce knee OA pain in a recent randomized, double-blind, sham-controlled, multicentre trial of 180 patients with mild-to-moderate knee OA. In that study, a single cryoneurolysis treatment of the infrapatellar branches of the saphenous nerve resulted in a reduction in pain and symptoms for at least 90 days [28]. Further, in a single-center, randomised controlled trial of pre-operative cryoneurolysis in patients scheduled to undergo primary unilateral total knee arthroplasty for OA, per-protocol analyses showed that compared to standard care, cryoneurolysis significantly reduced opioid consumption post-operatively [29]. Using cryoneurolysis in participants with ankle OA could provide immediate symptomatic relief with a greater duration of analgesia, and with a lower rate of side-effects than other non-operative treatments. Given the promising results for alleviating knee OA pain [28,29], combined with the limited therapeutic options for ankle OA, our aim was to evaluate the effectiveness of cryoneurolysis for improving ankle symptoms and function in participants with unilateral, symptomatic ankle OA.

## 2 Methods

### 2.1 Study design and recruitment

We conducted a single-center, single-arm open-label clinical trial with up to 24-weeks follow-up to examine the effectiveness of cryoneurolysis for reducing pain, improving activities of daily living (ADL) and quality of life (QoL) in patients with evidence of unilateral symptomatic ankle OA. We hypothesised that treatment of the superficial nerves and/or deep fibular nerve would result in significant improvements in function and pain at 24-weeks follow-up. This study was conducted at the University of Kansas Medical Center (Kansas City, KS, USA) from August 13, 2018 to January 26, 2021. Participants were assigned to undergo cryoneurolysis of either the Superficial Fibular Nerve (SFN), Sural Nerve (SN), and/or Saphenous Nerve, or the Deep Fibular Nerve (DFN) using ultrasound-guided cryoneurolysis. This study was performed in accordance with the provisions of the Declaration of Helsinki, and the protocol was approved by the University of Kansas Institutional Review Board (STUDY00142298). Written informed consent was obtained from all participants before inclusion. The trial was registered at clinicaltrials.gov (NCT03567187). Participants were identified through hospital records, physician referrals, mass mailings, and advertisements.

### 2.2 Study participants

Eligible participants were men and women aged >18 years, had radiographic evidence of ankle OA (Kellgren-Lawrence (KL) Grade ≥2; measured on weight-bearing mortise views with 20° internal rotation), were limited by unilateral ankle pain rated on a Numerical Rating Scale (NRS) as ≥5/10 on most days over the last month, had a Foot and Ankle Outcome Score (FAOS) [30] of <75 (0–100, 100 ​= ​worst possible pain) in at least 1 domain, body mass index (BMI) ≤50 ​kg/m^2^, were ambulatory and able to comply with study procedures, had undergone at least one prior conservative OA treatment (e.g. physical therapy, analgesics, ankle brace) and were willing to abstain from the use of protocol-restricted medications during the trial, and analgesics, other than acetaminophen, 1 week prior to the beginning of the trial.

Participants were excluded if they had another functional impairment that limited their walking ability to a greater extent than their ankle, had clinical signs/symptoms of active or recurrent infection in the index ankle joint (or overlying skin), intra-articular (IA) corticosteroids within 3-months of screening, oral corticosteroids within 2-weeks of screening (unless on a chronic stable dose for ≥3 months prior to enrolment), women who were pregnant, ankle pain due to a condition other than OA, arthroscopy or open surgery of the ankle joint within 6 months of screening, planned/anticipated surgery of the index ankle during the 6-month trial period or had a diffuse pain condition (e.g. diffuse pain including the bilateral upper and lower limbs; confounding pain such as knee, hip or back pain; or fibromyalgia). A full list of the exclusion criteria is included in Supplementary Table 1.

### 2.3 Study duration

**Fig. 1:**
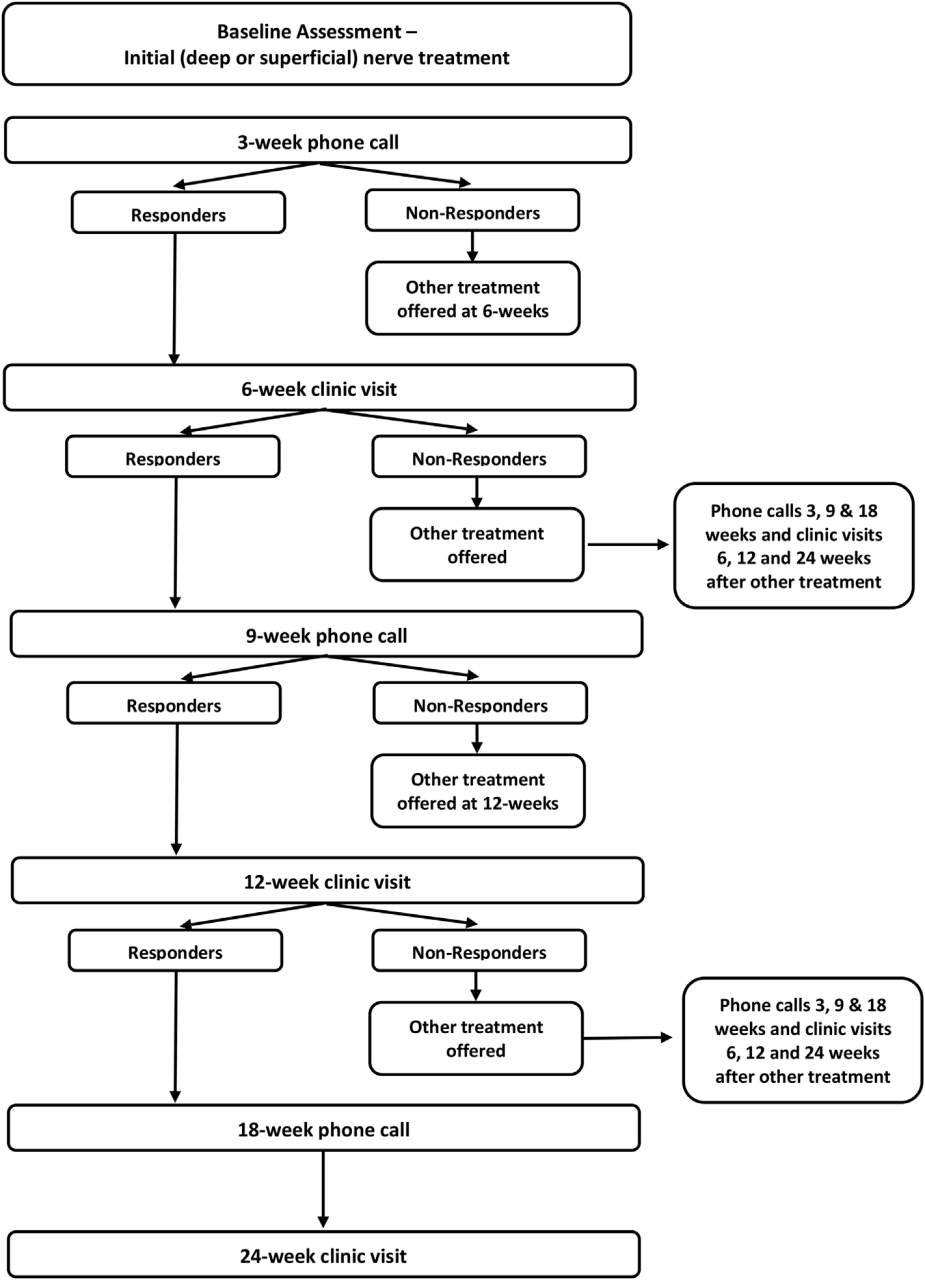
Protocol flowchart depicting treatment path based on criteria for responders (≥20% improvement in numeric rating score (NRS) pain from baseline) and non-responders ( <20% improvement in NRS pain from baseline) at each follow-up time point.

All participants were followed for up to 24-weeks following cryoneurolysis. Demographic and clinical characteristics for all participants were captured at baseline, 3, 6, 9, 12, 18 and 24-weeks follow-up. Fig. 1 depicts the flow of how participants were treated and offered an alternative treatment if their initial treatment failed to provide durable benefit (i.e. were identified as a ‘non-responder’).

‘Responder’ status was assessed using the NRS for pain at clinic visits (6-, 12- and 24-weeks post treatment) and via web-form or telephone interviews (as per the participants preference) at 3-, 9- and 18-week post treatment. Non-responders were defined as reporting <20% pain relief with respect to their baseline NRS pain score. Participants who were identified as non-responders to initial treatment were eligible to receive cryoneurolysis of the other nerve group at the next clinic visit. For example, if a participant was a non-responder 9 weeks after superficial nerves were treated, then at the 12-week clinical follow-up the participant would be offered treatment of the deep fibular nerve and if received, the 12-week visit would then become the new baseline visit for assessing outcomes of the new treatment.

### 2.4 Treatment & intervention

Depending on the location of the participants ankle pain, either the superficial nerves (sural nerve for lateral ankle pain, superficial fibular nerve for anterior ankle pain, and/or saphenous nerve for medial ankle pain) or the deep fibular nerve for deep ankle pain was treated with cryoneurolysis. A sonographically-guided diagnostic nerve block using 1% lidocaine with 1:100,000 epinephrine was performed for each nerve to confirm analgesia prior to treatment. Cutaneous sensation in the distribution of each nerve treated was assessed with a standardized nylon monofilament prior to and following cryoneurolysis. The anatomical locations of the respective nerves of interest and the cutaneous distribution tested are described below.

i) Superficial Fibular Nerve

The superficial fibular nerve (SFN) was sonographically located in the distal third of the leg at a location approximately 12 ​cm above the tip of the lateral malleolus, where it exited the fascia of the lateral compartment of the leg. Sensory function was tested on the dorsum of the foot.

ii) Sural Nerve

The sural nerve (SN) was sonographically located approximately 12 ​cm proximal to the posterior border of the lateral malleolus, and lateral to the Achilles’ tendon. Sensory function was tested on the lateral side of the ankle and fifth ray of the foot.

iii) Saphenous Nerve

The saphenous nerve was sonographically located approximately 7 ​cm proximal to the medial malleolus, and adjacent to the greater saphenous vein. Sensory function was tested just distal to the medial malleolus.

iv) Deep Fibular Nerve

The deep fibular nerve and anterior tibial artery were sonographically located by following the anterior surface of the tibia distally in transverse view. Generally, the deep fibular nerve was located lateral to the anterior tibial artery approximately 6 ​cm proximal to the ankle joint line. Sensory function was tested over the dorsum of the webspace between the first and second toes.

### 2.5 Cryoneurolysis device

The iovera° cryoneurolysis device (Pacira CryoTech, Inc, Fremont, CA, USA) is approved by the United States Food and Drug Administration (510(k) clearances K133453 and K161835), and is used in surgical procedures to alter nerve function by forming precisely controlled, sub-dermal cold zones. Exposure to localised temperatures of −20 to −80 ​°C temporarily disrupts peripheral nerve function by axonal and myelin degeneration known as Wallerian degeneration [26,27]. When sensory nerves are treated, their ability to convey sensory signals, such as pain, is immediately interrupted thereby producing an analgesic effect; this is followed by nerve functional restoration. Cryoanalgesia is an established principle and has been described previously [28,31]. The iovera° system uses liquid nitrous oxide (N_2_O) that is contained within the handpiece and no gas is injected into the body [26].

While the initial three participants were treated using a 6.9 ​mm 27G triple tip (model 307 between 8/13/2018–11/5/2018), most participants were treated with a 55 ​mm, 22-gauge single-point cryoprobe (model 155 between 12/3/2018–9/17/2020) due to the need for precise localization of the cryoprobe with continuous sonographic monitoring to avoid nerve damage. For each nerve treated, at least 4–8 cryoneurolysis cycles were completed (range: superficial nerves [3–12], deep fibular nerve [3–10]).

### 2.6 Assessments

At the screening/treatment visit, body mass index (BMI, kg/m^2^) was calculated from body mass (kilograms) divided by the square of the participant's height in meters (stadiometer, Holtain, Wales, UK); as measured by a trained member of the research team.

### 2.7 Outcome measures

The primary endpoint was change in ankle pain, assessed using the Foot and Ankle Outcome Score (FAOS), from baseline to 6, 12, and 24 weeks post-treatment respectively, with the 12-week outcome being the *a priori* primary study outcome. Based on animal studies, it is estimated that the rate of axonal regeneration following cryoneurolysis is approximately 1.0–3.0 ​mm/day [32,33]. Based on previously observed rates of recovery, although highly variable, we hypothesised a duration of pain relief in the ankle of approximately 90 days. Secondary outcomes included improvement in quality of life (FAOS-QoL subscale), activities of daily living (FAOS-ADL subscale) and Numerical Rating Scale (NRS) for pain. The tertiary outcome was change in physical performance on the 40-m fast paced walking test.

### 2.8 Patient-reported outcomes

I) Joint Symptoms, Activities of Daily Living and Quality of Life

Pain, ADL and QoL were assessed at baseline, 12 and 24 weeks post-treatment using the FAOS [30], a self-reported, region-specific questionnaire used for the assessment of foot and ankle joint health. The FAOS questionnaire comprises 42 items across 5 subscales of pain, other symptoms, ADL, sport and recreational function, and foot and ankle-related QoL [30] with a worst possible score of 0 and best possible score of 100. Pain severity was also reported using the NRS at baseline, 12- and 24-weeks post-treatment. Participants scored their ankle pain over the past 7 days from 0 to 10 (0 ​= ​no pain, 10 ​= ​worst pain imaginable).

### 2.9 Physical performance

Gait speed was measured in meters per second (m/sec) using the 40-m fast paced walking test (40 ​m FPWT). The 40m-FPWT is routinely used in clinical practice for the assessment of physical function and is one of the Osteoarthritis Research Society International (OARSI) recommended functional performance-based outcome measures for OA research [34].

### 2.10 Adverse events

Adverse events (AE) were systematically assessed throughout the study by phone and at each visit. In addition, participants were encouraged to contact the study team with information about AEs that occurred between each respective visit. When an AE was reported to the study staff, data including the type of event, onset/end dates, duration, severity, and outcome were collected and reported to the Principal Investigator (PI). The PI determined the severity of the event using the CTCAE version 5.0 guidelines [35]. Following treatment with cryoneurolysis, expected symptoms included numbness of the skin (ankle and/or foot) with possible skin redness, swelling, bruising or pain at the site of insertion.

### 2.11 Statistical analysis

To assess the primary hypothesis that cryoneurolysis reduced ankle pain for at least 12 weeks in people with unilateral, symptomatic ankle OA, a sample size was estimated based on published effect sizes of 1.06 for FAOS-pain and FAOS-QoL, and 0.65 for FAOS-ADL [36]. At an effect size of 0.65 and a single-sided alpha of 0.025, a sample size of 32 would provide a power of 90%. While 80% power is customary, this study was powered at 90% to reduce the probability of missing an effect if one was present, and to provide sufficient power for comparisons at 2-time points. Assuming up to 20% dropout, 40 participants were recruited. This sample size was considered to be more than sufficient since the effect size for the other 2 outcomes was reported to be larger at 1.06 [36].

The *a priori* primary analysis examined the change in FAOS-Pain between baseline and 12 weeks following cryoneurolysis. The analyses of secondary (i.e. FAOS-ADL, FAOS-QoL, NRS pain) and tertiary (i.e. 40m-FPWT) outcomes involved longitudinal data collected at baseline, 6, 12, and 24 weeks. All response variables were modeled using longitudinal mixed models. Each model was based on two main effects: a random subject effect, and a fixed time effect consisting of four levels (baseline and three follow-up time points). The Akaike information criterion was used to determine an appropriate variance/covariance structure for each model. Each hypothesis was tested by examining appropriate contrasts and estimated linear forms in the overall mean and the main effects for time, using an alpha level of 0.05 to determine statistically significant change. All analyses were participant-based, with one ankle per participant, and were conducted using “*PROC MIXED*” in SAS Version 9.4 (SAS Institute, Cary, NC) with results presented as least-square means (LS means) and 95% confidence intervals (95% CI).

## 3 Results

Forty-three potentially eligible participants were screened. Of these, two were found by radiographs or weight-bearing CT (WBCT) scan to not have tibiotalar OA (KL ​= ​0) and were excluded. In addition, one participant was excluded due to an unstable ankle that required bracing and opioid therapy. Subsequently, 40 participants with unilateral, painful ankle OA were recruited. Participants (50% women) had a mean ​± ​standard deviation (SD, range) age of 63.0 (12.8, range 28–84) years and a BMI of 31.7 (6.8, range 20.4–46.8) kg/m^2^. Most study participants localized their pain to the superficial nerve group. Five participants reported pain deep into the ankle joint for whom we performed deep fibular nerve treatment with cryoneurolysis. Thirty-one participants were initially treated with cryoneurolysis of superficial nerves (24 superficial fibular nerves, 29 sural nerves, 25 saphenous nerves) and 4 were initially treated at the deep fibular nerve. One participant who was initially treated at the deep fibular nerve was later treated in the superficial group and four participants who were initially treated with the superficial group later received a treatment of the deep fibular nerve. Baseline clinical and demographic characteristics of eligible participants are presented in Table 1. There were no obvious systematic differences in baseline characteristics between participants who completed 24 weeks of the study following initial treatment and those who either dropped out following the initial intervention or transitioned to the other treatment, except for a greater proportion of ankles graded as KL4 in those who dropped out vs. in all enrolled (12/18 vs. 19/40).

**Table 1:** Baseline demographics and clinical characteristics of eligible study participants and drop-outs.

Variable	Sample (N ​= ​40)	Drop-Outs (N ​= ​18)
Age (years), mean (SD, range)	63.0 (12.8, 28–84)	62.4 (13.3, 33–84)
Sex, n (% female)	20 (50)	11 (61.1%)
BMI (kg/m^2^)	31.7 (6.8)	29.0 (6.3)
Index ankle joint, n (% right)	27 (67.5%)	11 (61.1%)
KL grade of index ankle joint, n (%)		
***0***	2 (5.0%)∗	1 (5.6%)
***1***	0 (0.0)	0 (0.0)
***2***	5 (12.5)	1 (5.6)
***3***	14 (35.0)	4 (22.2)
***4***	19 (47.5)	12 (66.7)
NRS Pain Score, pain in past 7 days^a^	6.3 (1.6)	6.5 (1.9)
FAOS score^b^		
***FAOS-Pain***	43.1 (14.1)	41.3 (16.0)
***FAOS-ADL***	53.3 (16.4)	52.1 (17.8)
***FAOS-QOL***	22.0 (16.9)	22.8 (18.1)
Physical Performance		
***40-m fast-paced walking test (m/sec)***	32.8 (9.2)	33.2 (7.4)

Abbreviations: BMI, body mass index; SD, standard deviation; NRS, numeric rating score; FAOS, Foot and Ankle Outcome score; KL, Kellgren-Lawrence; ADL, activities of daily living; QOL, quality of life; m/sec, meters per second. ∗Following enrolment, one participant was found to have KL4 talonavicular OA and one was found to have subtalar joint OA, although they were enrolled with outside diagnoses of ankle OA.

All results are shown as means with standard deviations or, counts with percentages unless otherwise stated.

a Scores range from 0 to 10 with a score of 0 indicating no pain and 10 indication worst possible pain.

b Scores range from 0 to 100 with a score of 0 indicating the worst possible foot/ankle symptoms and 100 indicating no foot/ankle symptoms.

**Fig. 2:**
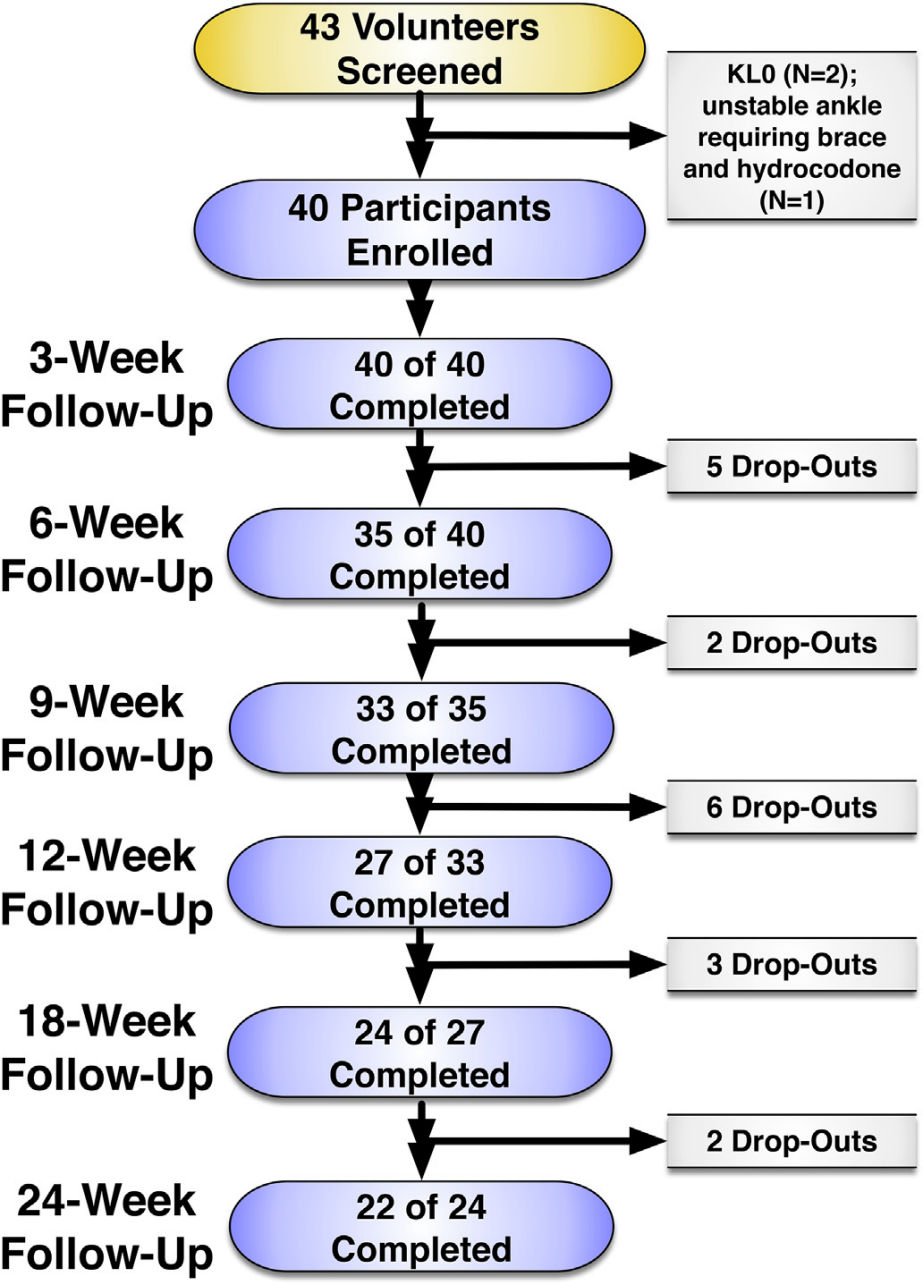
Participant flow at each study visit.

Twenty-two participants completed the full 24-week follow-up. Reasons for discontinuation are presented in Fig. 2. In brief, 7 participants discontinued due to increased pain after the intervention, 5 transitioned to the alternative treatment within the study per protocol, 2 sought treatment outside of the study (1 for repeat cryoneurolysis at Week 22 after study entry and 1 for an ankle corticosteroid injection at Week 18), 1 could not afford to travel for follow-up visits, and 3 did not answer calls or emails. The mean values for outcome measures at baseline and each follow-up visit are presented in Table 2.

**Table 2:** Least Square Means (95% CIs) for each outcome measure at each follow-up visit (p-values for comparison with baseline values).

Variables	Baseline	6 weeks	**p-value**	**12 weeks**	**p-value**	24 weeks	**p-value**
**FAOS-Pain**	43.1 (38.6–47.5)	60.5 (53.7–67.3)	< ​**0.0001**	63.9 (56.1–71.7)	< ​**0.0001**	64.8 (57.2–72.4)	< ​**0.0001**
**FAOS-ADL**	53.3 (48.2–58.4)	69.9 (63.2–76.5)	**0.0002**	71.4 (63.2–79.7)	**0.0003**	73.4 (67.0–79.8)	< ​**0.0001**
**FAOS-QoL**	22.0 (16.7–27.3)	44.5 (37.6–51.3)	< ​**0.0001**	41.9 (32.9–50.9)	**0.0003**	47.4 (39.6–55.3)	< ​**0.0001**
**NRS Pain over prior 7 days**	6.3 (5.8–6.8)	3.6 (2.8–4.5)	< ​**0.0001**	3.7 (2.7–4.8)	< ​**0.0001**	3.5 (2.6–4.4)	< ​**0.0001**
**40m-FPWT (m/sec)**	32.8 (29.9–35.7)	32.7 (29.3–36.1)	**0.9670**	31.6 (28.4–34.8)	**0.5911**	31.0 (26.8–35.1)	**0.4782**

Abbreviations: NRS, numeric rating score; FAOS, Foot and Ankle Outcome score; ADL, activities of daily living; QOL, quality of life; 40m-FPWT. 40-meter fast-paced walking test.

All results are shown as least square means with 95% confidence intervals (95% CI) unless otherwise stated.

### 3.1 Outcome measures

For the primary endpoint, there was a statistically and clinically significant improvement in FAOS-Pain from baseline to 12-weeks post-treatment (LS mean, 95% CI) of 20.8 (11.9, 29.8) points. Improvement was also detected in FAOS-Pain from baseline to 6- and 24-weeks, with 17.4 (9.3, 25.6) points and 21.7 (12.9, 30.5) points improvement respectively. For secondary outcomes, improvement was also found in FAOS-ADL, FAOS-QoL and NRS pain scores. For the FAOS-ADL subscale, a statistically significant improvement of 16.6 (8.2, 25.0) points was detected at 6-weeks, 18.1 (8.4, 28.0) points at 12-weeks and 20.1 points (11.9, 28.3) at 24-weeks. For the FAOS-QoL subscale, statistically significant improvement of 22.4 (13.8, 31.1) points was observed at 6 weeks, 19.9 (9.4, 30.3) points at 12 weeks, and 25.4 points (15.9, 34.9) at 24-weeks. Change in NRS pain score (LS mean; 95% CI) for the prior 7 days was also improved from baseline at 6-weeks (−2.7; −3.7, −1.7), 12-weeks (−2.6; −3.7, −1.4) and 24-weeks (−2.8; −3.9, −1.7) respectively. For the tertiary outcome, treatment was not associated with a statistically significant change in 40m-FPWT at any timepoint; see Table 2.

### 3.2 Safety

Over the 24-week follow-up period following cryoneurolysis, 19 participants reported a total of 42 adverse events. Of these, 2 were deemed to be unrelated to the intervention, 4 unlikely related, 18 possibly related, 14 probably related (paraesthesia for 6, pain in the treated ankle in 2, muscle cramp in 1, pruritis in 1, lump or edema in 2, and 1st web space pain in 1), and 5 definitely related (ankle pain immediately following the procedure in 3, a blister over the saphenous nerve treatment site in 1 and a bruise in 1). A full list of the reported adverse events is presented in Table 3. The most frequently reported adverse events were index ankle arthralgia (N ​= ​14) and paraesthesia (N ​= ​11), and most were of mild or moderate severity (81%). The 8 adverse events that were severe enough to limit sleep or self-care were ankle pain (N ​= ​6), foot muscle cramp (N ​= ​1) and pain in the 1st web space of the foot (N ​= ​1). There were no serious adverse events.

**Table 3:** Adverse events (AE) reported in eligible study participants.

Characteristic	Number of Occurrences (% participants)^a^
Arthralgia	14 (35%)
Bruising	2 (5.0%)
Bullous Dermatitis	1 (2.5%)
Fall	2 (5.0%)
Localized edema	5 (12.5%)
Muscle cramp	3 (7.5%)
Pain (1st web space)	1 (2.5%)
Paraesthesia	11 (22.5%)
Pruritus	1 (2.5%)
Skin Disorder - Other (lump)	2 (5.0%)
**Total**	**42**

A total of 19 participants reported N ​= ​42 adverse events.

a Adverse events were not mutually exclusive and study participants could report the same adverse event at different time points.

## 4 Discussion

This single-center, single-arm, open-label study is the first to evaluate the symptomatic and functional benefits of cryoneurolysis in participants with unilateral, symptomatic ankle OA. We observed statistically significant changes from baseline in FAOS subscales for pain, ADL, QoL, and in NRS-pain at 6, 12 and 24-weeks post-treatment. These data suggest that cryoneurolysis is likely to be effective to relieve pain and improve self-reported function in patients with symptomatic ankle OA, although walk time was not found to improve. These findings warrant further investigation and validation by future randomised, placebo-controlled trials.

Cryoneurolysis has been shown to be an effective treatment for decreasing knee pain [28], and improving post-operative outcomes following knee arthroplasty [29,37] yet the application of cryoneurolysis for ankle OA had not been studied. Cryoneurolysis is not expected to permanently reduce symptoms, as sensory nerves regenerate [27]. Unlike pharmacological treatments (e.g. NSAIDs, acetaminophen) which carry risk of side-effects [38,39], patients may undergo repeated cryoneurolysis to extend symptomatic benefits. In the current study and compared to baseline, statistically significant improvements in pain, ADL and QoL were observed at 6-, 12- (primary outcome) and 24-weeks following treatment. These effects are similar to a previous knee OA randomised trial in which, compared to sham, cryoneurolysis demonstrated statistically significant improvement in pain at days 30, 60 and 90 [28]. Further, WOMAC pain responders at day 120 continued to experience a statistically significant treatment effect at Day 150 (∼21.5 weeks) [28]. However, no statistically significant improvement in 40m-FPWT was observed despite finding a statistically significant improvement in ADL. These data suggest that despite improvements in functional ability and pain relief, patients with symptomatic, unilateral ankle OA may continue to be cautious or limited in walking.

In the current study, cryoneurolysis was well tolerated by most participants with a total of 42 adverse events (AEs) reported in 19 participants over the full 24-week study period. The most common AEs were arthralgia (N ​= ​14) and paraesthesia (N ​= ​11), which were mostly mild in severity and did not require clinical intervention. The higher occurrence of these AEs in the initial group of participants was attributed to the use of the 6.9 ​mm sharp tips and was very rare after changing to 55 ​mm dull tips that allowed continuous sonographic guidance to avoid direct contact with the nerve. Even with sonographic guidance, it was thought that the use of the sharp tips may increase the risk of puncturing the nerve sheath leading to increased post-cryoneurolysis dysesthesias observed in the first three participants treated. After switching to the 55 ​mm tip, the procedure had substantially fewer AE's.

When compared to a previous study of cryoneurolysis in patients with mild-to-moderate knee OA [28], we reported similar counts of AEs. Whilst we did not have a sham group, in the same study, the incidence of device- or procedure-related AE were similar across treatment and sham arms [28] giving confidence to the safety of the treatment. Our rates of AEs were significantly fewer compared with the rates reported for standard of care therapy (i.e. corticosteroid injection) [40]. These data suggest that cryoneurolysis may provide comparable or superior pain relief with functional improvement with relatively low risks compared with currently available pharmacological treatments for OA.

One limitation of this study was that, upon re-assessment of the baseline radiographs following treatment, one participant had KL grade 4 talonavicular OA and one had subtalar joint OA, rather than tibiotalar OA. Given that these participants were treated for ankle pain and returned for follow-up, they were not excluded. Further, we used an open-label design without a sham treatment as this was the first study of cryoneurolysis for ankle OA, and the funding source elected to determine the magnitude of effect of the novel protocol used prior to initiating a controlled study. Large placebo effects have been observed in OA clinical trials, particularly in studies of surgical intervention [41]. While it is possible that the independent treatment effects of cryoneurolysis could be better evaluated by comparison against a sham intervention, the robustness of the response for at least 24 weeks in participants following years of severe ankle pain lends credibility to the study findings. These findings, however, require validation in future randomised, placebo-controlled trials. Cryoneurolysis is a palliative treatment approach and does not target the underlying cause of pain generation. Lastly, whilst 40 participants were recruited, only 22 participants completed the full 24-week follow-up period. Strengths of this study included the frequency of follow-up assessments and the duration of follow-up, design features that permitted measurement of pain, function, and quality of life following this novel application of cryoneurolysis.

## 5 Conclusions

Cryoneurolysis of the superficial and/or deep nerves surrounding the ankle resulted in significant improvements in ankle pain, function and QoL for up to 24-weeks in participants with unilateral, symptomatic ankle OA. These data support the use of cryoneurolysis as an effective and safe non-pharmacological treatment of joint symptoms in patients with symptomatic ankle OA.

## Role of funding source

Funding for this study, equipment and cryoprobes were provided by Pacira CryoTech, Inc. (previously Myoscience) through an Investigator-Initiated Research (IIR) unrestricted grant. The funders were not involved in the study design, data collection and interpretation of study results.

## Role of funding source

Funding for this study, equipment and cryoprobes were provided by Pacira CryoTechh, Inc. (previously Myoscience) through an Investigator-Initiated Research (IIR) unrestricted grant. The sponsor was not involved in the study design, data collection, data analysis, data interpretation or manuscript preparation. HERON is supported in part by funds from 10.13039/100016220CTSA Award #UL1TR000001and 10.13039/100006093Patient-Centered Outcomes Research Institute (10.13039/100006093PCORI) Program Award #CDRN-1306-04631.

## Author contributions

Conception, design and conduct of study: NAS. Analysis and interpretation of data: both authors. Drafting Article: both authors. Critical revision of article: both authors. Final Approval: both authors.

## Availability of data and materials

All data generated and analysed in this study are available upon reasonable request. Access to data generated in this report should be sent to the corresponding author at thomas.perry@kennedy.ox.ac.uk.

## Public and patient involvement (PPI) statement

PPI was not required nor involved with any aspect of the work presented.

## Ethical approval

Ethical approval was granted by the 10.13039/100007859University of Kansas Institutional Review Board (STUDY00142298).

## Declaration of competing interest

Neither author declares conflicts of interest related to the current study. NAS has consulted for Flexion Therapeutics for unrelated work.
