# Supplementary material for: An open-label, single-arm trial of cryoneurolysis for improvements in pain, activities of daily living and quality of life in patients with symptomatic ankle osteoarthritis
Source: Osteoarthr Cartil Open. 2022 May 15;4(3):100272. doi: 10.1016/j.ocarto.2022.100272 (PMC9718236; doi:10.1016/j.ocarto.2022.100272)
Supplement: Multimedia component 1 [file mmc1.docx]

| **Supplementary Table 1 -** Study Exclusion criteria. |
| --- |
| - Baseline knee, hip, spine or other limitations that affect the walking ability to a greater extent than their ankle - Cryoglobulinemia, paroxysmal cold hemoglobinuria, Raynaud’s disease, cold urticaria. - Clinical signs or symptoms of active or recurrent infection in the index ankle joint or overlying skin - IA corticosteroid (investigational or marketed) within 3 months of screening. - Oral corticosteroids (investigational or marketed) within 2 weeks of screening (unless on a chronic stable dose for ≥3 months prior to enrolment) - Pregnant (due to potential for the change in body mass and distribution to alter ankle symptoms over the period of follow-up) - Any condition other than OA of the ankle joint which, in the opinion of the investigators, affects their ability to ambulate to a sufficient degree or interferes with the assessment of the safety and treatment effects of the study injection - Arthroscopy or open surgery of the ankle joint within 6 months of screening - Planned/anticipated surgery of the index ankle joint during the 6-month study period - Skin breakdown at the ankle joint where the injection is planned to take place. - Participated in any investigational drug or device trial within 30 days prior to screening or concurrent participation in another research study that could complicate interpretation of the findings of either study - Current consumption of more than 14 alcoholic drinks per week - Patients with diffuse pain conditions - Known altered nerve anatomy or physiology (e.g. neuropathy) at the target, such as due to a congenital, traumatic, medical or surgical cause |
| **Abbreviations:** Intra-articular (IA). |
